# Integrating 3D structural information into systems biology

**DOI:** 10.1016/j.jbc.2021.100562

**Published:** 2021-03-18

**Authors:** Diana Murray, Donald Petrey, Barry Honig

**Affiliations:** 1 Department of Systems Biology, Columbia University, New York, New York, USA; 2 Department of Systems Biology, Department of Biochemistry and Molecular Biophysics, Department of Medicine, Zuckerman Mind Brain and Behavior Institute, Columbia University, New York, New York, USA

**Keywords:** systems biology, protein structure, protein–protein interaction, homology modeling, computational biology, HT, high-throughput, ML, machine learning, PDB, Protein Data Bank, P-HIPSTer, Pathogen Host Interactome Prediction using structure similarity, PPI, protein–protein interaction, PrePPI, Predicting Protein-Protein Interactions, TCGA, The Cancer Genome Atlas

## Abstract

Systems biology is a data-heavy field that focuses on systems-wide depictions of biological phenomena necessarily sacrificing a detailed characterization of individual components. As an example, genome-wide protein interaction networks are widely used in systems biology and continuously extended and refined as new sources of evidence become available. Despite the vast amount of information about individual protein structures and protein complexes that has accumulated in the past 50 years in the Protein Data Bank, the data, computational tools, and language of structural biology are not an integral part of systems biology. However, increasing effort has been devoted to this integration, and the related literature is reviewed here. Relationships between proteins that are detected *via* structural similarity offer a rich source of information not available from sequence similarity, and homology modeling can be used to leverage Protein Data Bank structures to produce 3D models for a significant fraction of many proteomes. A number of structure-informed genomic and cross-species (*i.e.*, virus–host) interactomes will be described, and the unique information they provide will be illustrated with a number of examples. Tissue- and tumor-specific interactomes have also been developed through computational strategies that exploit patient information and through genetic interactions available from increasingly sensitive screens. Strategies to integrate structural information with these alternate data sources will be described. Finally, efforts to link protein structure space with chemical compound space offer novel sources of information in drug design, off-target identification, and the identification of targets for compounds found to be effective in phenotypic screens.

The growth of protein structure information has stimulated a parallel growth in computational tools that predict protein structure and function. These tools provide fundamental insights into the physical principles that underlie the behavior of biological macromolecules. For example, molecular dynamics simulations allow realistic descriptions of conformational heterogeneity; Poisson–Boltzmann calculations have revealed how electrostatic interactions play a central role in biological functions; and the forces that determine the stability of the native folded state are now well understood. Advances such as these have been transformative and are part of the language and intellectual foundation of modern structural biology.

A parallel set of computational methods falls under the rubric of “structural genomics,” which includes the goal of structurally characterizing enough members of sequence families so as to enable the construction of homology models for the others. A key development has been the computational identification of geometric relationships among protein structures. Since structural similarity can identify functional relationships even in the absence of statistically significant sequence similarity, structural alignment has become a powerful tool to detect evolutionary relationships between proteins that cannot be detected from sequence alone. We have used the term Structural Blast (1) to imply the use of structural alignment to identify relationships between proteins in analogy to the widely used BLAST suite of programs for sequence alignment (2). Figure 1 provides two examples of functional relationships that can be detected this way: protein–protein interaction (PPI) and protein–compound interaction. Figure 1*A* illustrates the structural alignment of four protein domains where BLAST fails to detect any sequence relationship between them. Figure 1*B* shows the experimentally determined complex between the pleckstrin homology (PH) domain from phospholipase C-gamma-2 (*yellow*) and the small GTPase Rac2 (*gray*). Structural alignment of the Ezrin F3 lobe (*red*) with the PH domain produces a model for the complex between Ezrin and Rac2 (*red*–*gray*). Similarly, Figure 1*C* shows the experimentally determined complex between the PH domain from mouse Beta-II spectrin (*green*) and inositol 1,4,5-trisphosphate (*sticks*). Structural alignment of the Tiam-2 PH domain (*blue*) with the Beta-II spectrin PH domain produces a model for the complex between Tiam-2 and inositol 1,4,5-trisphosphate (*blue* and *sticks*). These examples provide the basis of many of the methods highlighted later that, as will be described, enable the use of structural information on a genomic scale.

**Figure 1:**
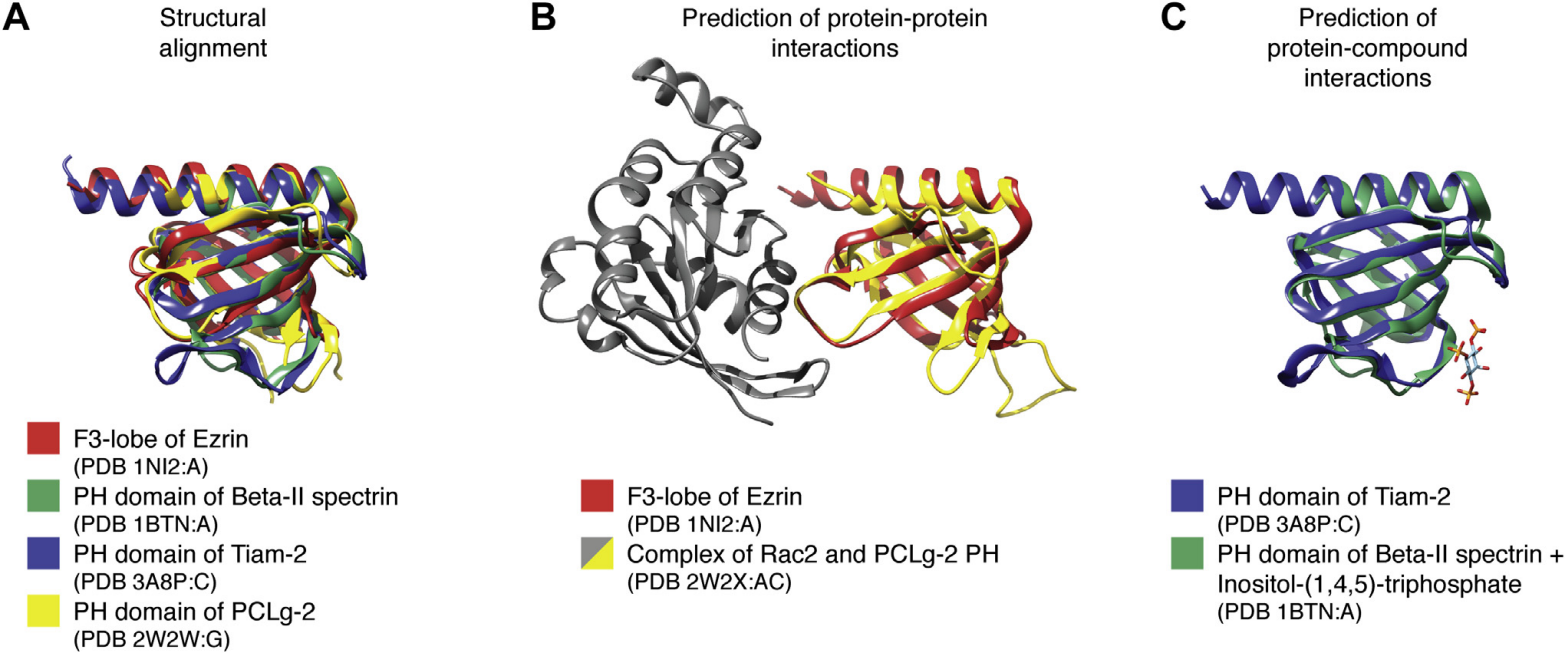
**Detecting protein–protein and protein–compound interactions with Structural Blast.***A,* F3 lobe of Ezrin from Protein Data Bank (PDB) 1ni2:A (*red*); pleckstrin homology (PH) domain of Beta-II spectrin from PDB 1btn:A (*green*); PH domain of Tiam-2 from PDB 3a8p:C (*blue*); and PH domain of PLCg-2 from PDB 2w2w:G (*yellow*). *B,* structure alignment of F3 lobe of Ezrin (1ni2:A, *red*) to the PDB complex (2w2x:AC) of Rac2 (*gray*) and PLCg-2 PH (*yellow*). PrePPI (35) uses this template complex to predict an interaction between Rac2 and Ezrin. *C,* the first PH domain of Tiam-2 is predicted by LT-Scanner (94) to bind inositol-(1,4,5)-triphosphate based on structure superposition of a homology model for Tiam-2 (*blue*) onto the PH domain of Beta-II spectrin (*green*) complexed with inositol-(1,4,5)-triphosphate (*stick representation*) (PDB: 1btn). Even though the sequence identity between the proteins is undetectable by pairwise global sequence alignment, the five residues specifically involved in binding inositol-(1,4,5)-triphosphate (102) are largely conserved in Tiam-2 as revealed by structure-based sequence alignment (K–K, R–R, S–R, Y–H, K–K, W–W).

The Protein Data Bank (PDB) (3) stands as a centerpiece of structural biology. It has created standards that impact the entire community, organized data in easily accessible form, and provided a battery of tools and links to other databases that have revealed multiple ways in which 3D structural information can be exploited for the detailed annotation of protein function and interactions. Indeed, much of the research that is discussed here would not have been possible without extensive use of the PDB and its many auxiliary resources.

There are areas of biomedical research where protein structure is still underutilized. Specifically, cellular systems biology, with its heavy emphasis on the study of pathways and networks, has made only limited use of 3D information. In networks, PPIs are typically described as nodes (proteins) connected by edges (interactions), without reference to the structures of the proteins involved or the nature of the interactions. With 20,000 human protein coding genes and potentially millions of PPIs, it is not possible to obtain experimental structures for every node and edge in the interactome. Computational methods to interrogate these interactions can complement the available experimental evidence, enabling more meaningful insights from systems biology approaches.

This article summarizes some of the advances in structural systems biology and points to strategies through which structural information can be integrated with the vast quantities of data emerging from high-throughput (HT) genomic technologies and patient records (summarized in Table 1). There are a number of computational methodologies that are central to this integration. First, the ability to construct homology models for most proteins in a given genome implies that, in principle, structure can be used on a genome-wide scale. Homology models dramatically enhance structural genomics efforts; for example, while there are structures available for about 5000 human proteins in the PDB, there are homology models for at least one domain of about 18,000 human proteins in databases such as ModBase (4) and SwissModel (5).

**Table 1:** Intersections between structural biology and systems biology

Systems level	Insight from computational structural biology
Protein	Models of protein domains (4, 5)
	Delineation of intrinsically disordered regions (97)
	Prediction of interaction surfaces (38, 94)
	Context of missense mutations (60, 98)
PPIs (33, 34, 35)	Determination of direct *versus* indirect
	Domain-level models of protein regions involved
	Atomic-level detail of interfaces
Pathways/networks	Molecular mechanisms for information flow
	Molecular depiction of complexes and series of PPIs
	Pathway/submodule crosstalk
	Hypothesis generation for effects of perturbations
	Rational targeting to alter phenotypic outcome (75)
	Integration with subcellular localization (99)
Tissue/tumor	Integration with context-specific data (27)
	Differential pathways/networks (100)
	Models for protein-mediated cell–cell interactions (101)

A second methodology has been the use of Structural Blast, as illustrated in Figure 1. The structure-based identification of a large number of functional relationships combined with extensive structural coverage of multiple genomes with homology models enables the prediction of PPIs on a genomic scale. Third, machine learning (ML) is crucial to the integration of structural and genomic data. ML not only facilitates the combination of data from multiple sources but also mitigates inaccuracies in structural models since training will determine the extent to which the models have predictive value. In this regard, it is important to emphasize that inferences yielded in systems biology are often statistical in nature, and the use of structural information must be used in such a way so as to conform to this reality.

This article is not meant as a comprehensive review of the literature, and many substantial studies do not appear on the reference list. Rather, our goal is to convey our own perspective of the development of a new interdisciplinary field and highlight articles that provide useful examples along with access to a larger literature. Our perspective is also embodied in our own contributions, some of which are summarized later.

## PPIs

The discovery and analysis of PPI networks has become an important area of systems biology where a particular focus has been specific applications to human disease. In systems-based approaches, genes or proteins are identified as disease associated based on their topological location in interaction networks (6, 7, 8). A necessary step in the creation of a network is the identification of interactions among proteins, which may include formation of stable dimeric or multimeric complexes; transient engagements that in some cases may be of low affinity and in others may involve post-translational modification; nonphysical interactions where, for example, one protein may regulate the expression of another in the absence of any physical contact between the two. It is necessary to keep these distinctions in mind when reading the PPI literature.

Given the centrality of PPIs in so many cellular processes, their experimental detection and computational prediction constitute a major research focus. Only HT experimental methods and highly efficient computational approaches are capable of detecting/predicting PPIs on a genomic scale. Complicating the challenge is the fact that physiological PPIs are context dependent: two proteins found to interact in an *in vitro* assay may well form a complex if expressed at appropriate levels but may never actually encounter one another *in vivo*.

### Databases of experimentally observed PPIs

There are many genome-wide PPI databases for human and different model organisms (9). Some are based on HT methods, such as yeast two-hybrid (10) and tandem affinity purification mass spectroscopy (11), whereas others are based entirely on literature curation (*e.g.*, BioGRID (12), IntAct (13), MINT (14)). Databases such as HINT (15), HURI (16), and APID (17) curate these resources to provide high-quality interactions and/or to extract only binary or physical associations. The widely used STRING database (18) combines literature curation with predictions based primarily on sequence relationships. With few exceptions, existing databases do not include context-specific information, such as the cell line, tissue, tumor type, disease condition, and others, in which the interactions are observed.

Context-specific associations can be derived from methods based on the correlation of gene profiles across many conditions (*e.g.*, cell lines or drug treatments) (19, 20). These profiles are typically obtained from HT genomic screens of cancer cell lines or human tissue samples: Project Achilles for RNAi and CRISPR–Cas9 knockdowns (21, 22); the Library of Integrated Network-Based Cellular Signatures (LINCS) (23) and the Cancer Dependency Map (CMap) (24) for phenotypic drug screens; The Cancer Genome Atlas (TCGA) for tumor-specific genetic variation (25); and Genotype-Tissue Expression (GTEx) for nondiseased tissue-specific genetic variation (24). The Califano laboratory has pioneered the use of algorithms to predict tumor-specific regulatory interactions based on the analysis of large-scale molecular profile data taken, for example, from TCGA (26). As will be discussed later, the integration of patient-specific regulatory networks with predicted physical interactions between proteins enables the development of context-specific structure-informed protein interaction networks, thus providing mechanistic insights not available from resources mentioned previously (27).

### Structure-informed prediction of PPIs in the human proteome

PPI prediction can involve (a) predicting the structure of known complexes given the structures of interacting monomers; (b) predicting whether and how two proteins interact given their structures, which requires building a model of the putative complex and then scoring it; (c) predicting whether two proteins interact given their sequence, which can be accomplished either by purely sequence-based methods, that is, sequence relationships to proteins in known complexes, or through some combination of methods (a) and (b). There are two main computational approaches for method (a): docking and template-based modeling. Docking methods (28, 29) are widely used but have not reached the point in terms of computation time where they can truly be used for genome-scale interactomes. Template modeling (30) involves superimposing the structures of two query proteins on structurally similar interacting proteins in a PDB complex (*e.g.*, Fig. 1). Algorithms to find such structurally related proteins are currently quite efficient (31, 32).

The Interactome3D server was an early resource for the prediction of the structures of protein complexes for different organisms (33). The current release lists binary interactions taken from experimental databases and, where possible, structural models for 18 organisms. Structures of complexes are obtained from either the PDB or template-based modeling with templates identified based on sequence relationships. For the human proteome, structural models are provided for ~15,000 binary complexes involving ~10,000 proteins; about half of the complexes are taken from the PDB. Overall, Interactome3D lists 125,000 experimentally observed binary PPIs for the human proteome with structural models for 12%.

Interactome INSIDER (34) also builds models for experimentally determined binary interactions. It is based in part on the Ensemble Classifier Learning Algorithm to predict Interface Residues (ECLAIR) framework, which combines features derived from individual proteins, such as surface properties, with pairwise PPI features obtained from docking and coevolution analysis. ECLAIR is trained on high-quality experimental data sets of PPIs (15). The current version contains over 120,000 predictions of structurally resolved interfaces for experimentally observed human PPIs. The high structural coverage of Interactome INSIDER is achieved by the use of docking, which avoids the necessity of a binary complex as a structural template; that is, only the structures of individual interacting proteins are needed.

**Figure 2:**
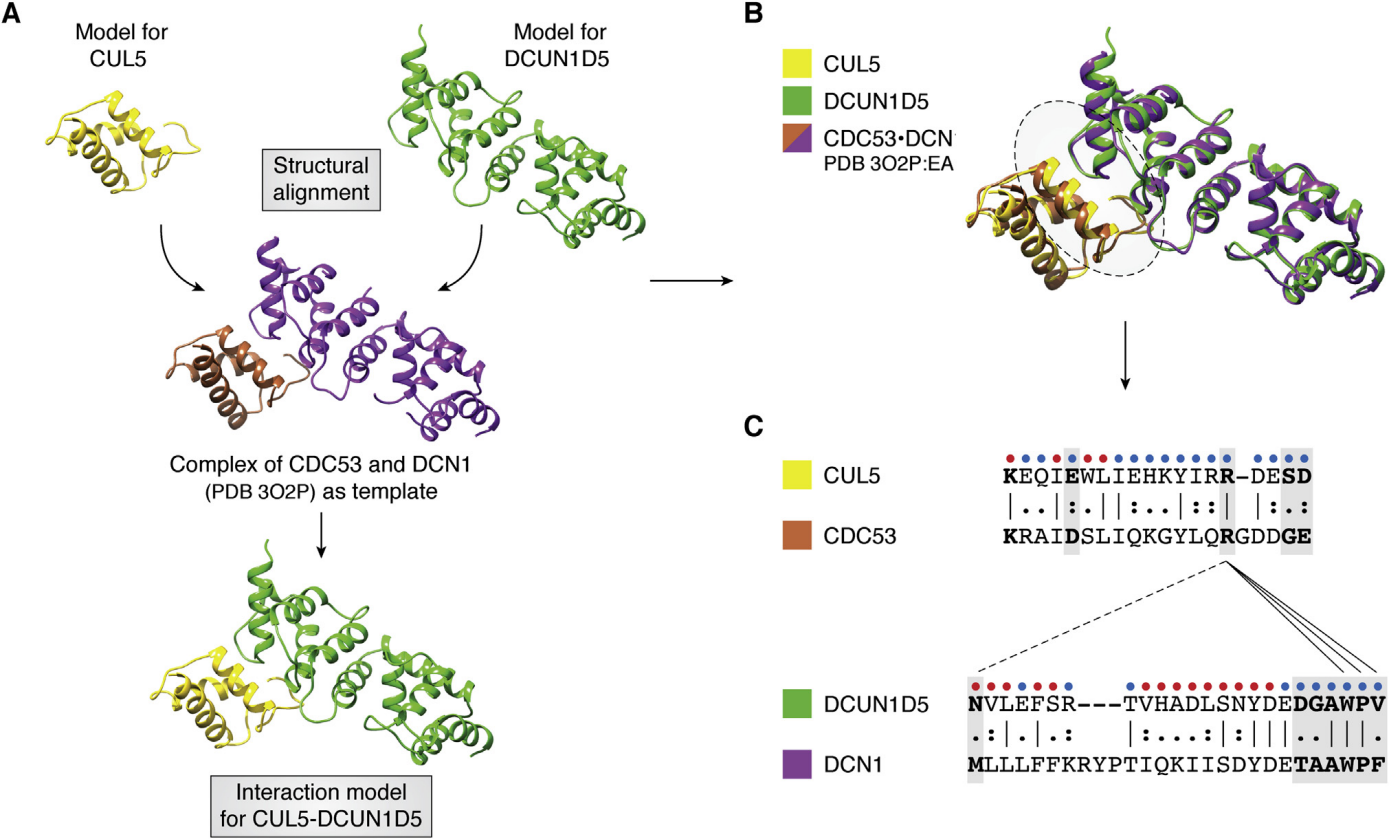
**Scoring protein–protein interaction (PPI) models with PrePPI.***A,* creation of a model (*yellow*–*green*) for the interaction between human Cullin-5 (CUL5) and defective in cullin neddylation protein 1 (DCN1)-like protein 5 (DCUN1D5) based on structural alignment of models for CUL5 (*yellow*) and DCUN1D5 (*green*) to the PDB template 3o2p:EA complex between the yeast cell division control protein 53 (CDC53; 3o2p, chain E, *brown*) and DCN1 (3o2p, chain A, *purple*). In spite of low sequence identities—23% for CUL5 *versus* CDC53, and 25% for DCUN1D5 *versus* DCN1—the models for CUL5 and DCUN1D5 align well to the template chains with low protein structure distances (32) of 0.05 and 0.04. The average of the protein structure distance values contributes to the PrePPI structure modeling (SM) score. *B,* structure superposition of models for CUL5 (*yellow*) and DCUN1D5 (*green*) on the PDB template 3o2p:EA. The *dotted circle* highlights the interaction interface, a portion of which is described in (*C*). *C,* structure-based sequence alignments for portions of the interface (*dotted circle* in (*B*)): CUL5 (*yellow*) on CDC53 (3o2p:E, *brown*), and DCUN1D5 (*green*) on DCN1 (3o2p:A, *purple*). The interaction model (*yellow*–*green*) is not explicitly scored; rather its relationship to the template (*brown*–*purple*) is evaluated for the PrePPI SM score. Positions of residues that make interfacial contacts in 3o2p:EA (*i.e.*, between CDC53 and DCN1) are bolded and boxed. The alignments reveal that all the residues involved in the template interface are aligned to residues in the query proteins, and this yields a favorable contribution to the SM score. Then, the propensity for these model residues to be interfacial is evaluated. PredUs (38) predicts whether residues in CUL5 and DCUN1D5 are interfacial (*blue*) or not (*red*). The contacts observed for R804 in the template are shown as *lines*: The *solid lines* denote the cases where the contacting template residues are aligned to residues in CUL5 and DCUN1D5 that are predicted to be interfacial (*blue dots*); the *dotted line* denotes the case where contacting template residues are aligned to model residues that are not predicted to be interfacial (*red dots*). Only those contacts that are likely to occur between the proteins in the interaction model (*solid lines*) further increase the SM score.

The Predicting Protein-Protein Interactions (PrePPI) algorithm is fundamentally different from Interactome3D and Interactome INSIDER in that it makes structure-informed predictions of whether two proteins interact independent of whether they appear in experimental databases (35, 36). Furthermore, PrePPI uses structure on a truly genome-wide scale, effectively screening most of the ∼200 million possible human PPIs. Like other methods, it begins with a database of ∼18,000 PDB structures and homology models for proteins and their constituent domains. PrePPI then uses structural alignment to establish relationships among protein structures: every one of the ∼18,000 query proteins is assigned a set of “structural neighbors” derived from structure alignments to protein structures in the PDB, regardless of species. Each query protein will have, on average, hundreds of neighbors. This large number results both from the use of distant structural relationships in multiple genomes and from the fact that the PrePPI alignment procedure defines neighbors when as few as three secondary structure elements can be aligned. If any two query proteins have neighbors that interact in the same PDB file (templates), then each of the query proteins is superimposed on its appropriate neighbor to generate a structural model for the interaction between those two query proteins. This is illustrated in Figure 2*A*, where models of the proteins for human CUL5 (*yellow*) and DCUN1D5 (*green*) are superimposed on chains of the PDB complex between the yeast proteins for CDC53 (*brown*) and DCN1 (*purple*). In this case, the template complex was identified because the proteins for human CUL5 and yeast CDC53 are structural neighbors as are the proteins for human DCUN1D5 and yeast DCN1.

The use of structural alignment in this way generates an extensive set of PPI models that are quickly scored by a naïve Bayesian ML algorithm, trained on experimentally determined PPIs. Scoring is a unique feature of PrePPI. Since hundreds of millions of interaction models are generated, some of them quite crude, applying standard energy functions would be computationally prohibitive. The approach used to enable the scoring of so many models is to transform the problem to one where pairwise information for the modeled interface (Fig. 2*B*) is transferred directly from the template interface (Fig. 2*C*). PrePPI scoring is based on the quality of the structural alignment of each individual protein to its template and on features of the alignment of query residues to interfacial residues in the template (37, 38); see figure legend for details. A likelihood ratio is calculated for each interaction, and a cutoff is defined for a “high-confidence” prediction.

PrePPI not only relies on structural information but also calculates likelihood ratios for nonstructural evidence such as whether the two query proteins have a similar function and whether their orthologs interact in other species, are coexpressed, or have a similar phylogenetic history (35). Nonstructural sources of evidence can increase the probability that a structural signal is real but can also have the effect of detecting interactions that are indirect. Overall, PrePPI performance at recovering known (gold standard) PPIs is comparable to that of other large-scale PPI databases and is comparable in accuracy to HT experimental methods (35). At present, the PrePPI database contains high-confidence predictions for over 1.3 million human PPIs where about 500,000 are predicted to be binary physical interactions. Many of these predictions are novel since the use of 3D structure detects many relationships that are not detectable with sequence. Of the 500,000 binary predictions, about 75% are predicted to be domain–domain interactions and 25% are predicted to be protein–peptide interactions. High confidence is of course a vague term, and indeed, PrePPI undoubtedly contains many false positives despite its overall success rate. However, it represents an attempt to replace sequence relationships with structural relationships on a genomic scale and, in doing so, generates testable hypotheses not available from other approaches. Of note, the approximately 800,000 PPIs that are not predicted to involve physical interactions likely involve proteins that are present in the same complex or participate in the same pathway but are not in direct contact.

There has also been major progress in the use of sequence-based approaches that exploit coevolution relationships to predict PPIs (39, 40). For the most part, these techniques require multiple sequence alignments of many orthologs and are, thus, largely limited to bacterial proteomes. Recently, Cong *et al*. (41) developed a hybrid approach to predict PPIs for the *Escherichia coli* proteome that first used coevolution to filter 4 million pairs of query protein sequences and then implemented docking with structures and homology models of the query proteins to produce a set of 800 predicted PPIs. Indeed, the combination of structural and coevolution information offers numerous strategies to predict PPIs, and there are likely to be exciting developments in this area in the coming years.

### Structure-informed prediction of virus/host PPIs

Viruses deploy an array of genetically encoded strategies to co-opt host machinery and support viral replicative cycles. Molecular mimicry, manifested by structural similarity between viral and endogenous host proteins, allows viruses to harness or disrupt cellular functions including nucleic acid metabolism and modulation of immune responses. Mimicry relationships have been detected through sequence similarity and linear motif co-occurrence (42, 43); however, structural similarity enables identification of mimics between pathogen and host proteins that cannot be observed from sequence alone (44). Structural mimicry can occur at the level of entire protein domains or in the form of “interface mimicry,” where the structure of host protein residues involved in PPIs is mimicked on the surface of a viral protein (45, 46, 47). Indeed, analysis of PDB structures has demonstrated that the interfaces in complexes involving a viral and human protein mimic the interfaces of human PPIs (48), and interface mimicry has been used as a basis for predicting virus/host PPIs (49, 50).

A recent study reported a systematic analysis of molecular mimicry across the entire virome (51). Protein structure similarity was used to scan for viral structure mimics from thousands of catalogued viruses and hosts spanning broad ecological niches and taxonomic range, including bacteria, plants and fungi, invertebrates, and vertebrates. The results point to molecular mimicry as a pervasive strategy employed by viruses and indicate that the protein structure space used by a given virus is dictated by the host proteome. In particular, analysis of the proteins mimicked by human-infecting viruses points to broad diversification of cellular pathways targeted *via* structural mimicry, identifies biological processes that may underlie autoimmune disorders, and reveals virally encoded mimics that may serve as targets for therapeutics.

Viral mimicry and, in particular, interface mimicry, indicate that viral proteins compete with host proteins for host interaction partners and, indeed, it is clear that knowledge of virus/host PPIs is critical for understanding mechanisms of infection. The PrePPI computational pipeline was used to create the Pathogen Host Interactome Prediction using structure similarity (P-HIPSTer) database (50). P-HIPSTer employs structural information to predict 282,000 pan viral–human PPIs with an experimental validation rate of 75% comparable to what was found for PrePPI for human PPIs (36). In addition to rediscovering known biology, P-HIPSTer has yielded a series of new findings: the discovery of shared and unique machinery employed across human-infecting viruses; a likely role for interactions between Zika Virus proteins and human Estrogen Receptor 1 in modulating viral replication; the identification of PPIs that discriminate between human papilloma viruses with high and low oncogenic potential; and a structure-enabled history of evolutionary selective pressure imposed on the human proteome. Furthermore, P-HIPSTer enables discovery of previously unappreciated cellular circuits that act on human-infecting viruses.

## Disease driver mutations and PPI networks

There has been enormous interest in understanding the role of mutations in disease, and 3D structural information has played an important role in this process. Much effort has been invested in the study of somatic mutations identified in the sequenced genomes of tumors and normal tissue available in resources such as TCGA (25) and the International Cancer Genome Consortium (ICGC) (52). There are tens of thousands of somatic mutations present in these genomes, and a major focus has been to identify “driver genes” that contain mutations capable of effecting tumorigenesis. Driver genes were initially identified as containing more mutations than expected from the background mutation rate, but the distribution of mutations on a particular protein also provides an important signal. Given that most tumors contain a large number of unique mutations, it has been necessary to develop sophisticated bioinformatics tools to analyze patient samples. These have focused on the identification of oncogenic “driver mutations” that are generally distinguished from “passenger mutations” that have no oncogenic potential. These classifications are somewhat ambiguous since a single driver mutation is not necessarily sufficient to cause cancer, whereas some passenger mutations might well be oncogenic when present along with other mutations or in specific contexts. The reader is referred to the excellent review by Martinez-Jimenez *et al*. (53) for an illuminating historical discussion of the large literature in the field.

In another insightful review, Porta-Pardo *et al*. (54) summarized algorithms that have been developed to identify driver genes based on the distribution of mutations they present. Some algorithms look for clusters of mutations along a protein sequence, whereas others identify clusters within a 3D structure (55, 56, 57); however, such approaches do not necessarily reveal mechanistic insights. Observations that disease mutations are enriched in protein–protein interfaces (58) suggest that cancer driver mutations can be identified on this basis. Indeed, mapping of somatic mutations obtained from TCGA onto PPI interfaces taken from the PDB and high-quality homology models identified about 100 interfaces enriched in somatic mutations involving proteins not previously identified as cancer drivers (59). In a landmark study, Bailey *et al*. (60) combined 26 computational tools, including some that were structure based, to classify about 750,000 pan-cancer missense mutations and identified 299 driver genes and over 3400 driver mutations. The information and mechanistic insights obtained from these studies are unique but perhaps limited by their focus on individual proteins. Algorithms that treat mutations as perturbations of both the nodes and edges in networks have been successful at annotating disease-associated genes and mutations (8). The integration of structural information into network biology is thus likely to yield important new insights into the identification of driver genes and molecular mechanisms underlying tumorigenesis.

## Adding context to interactome analysis

**Figure 3:**
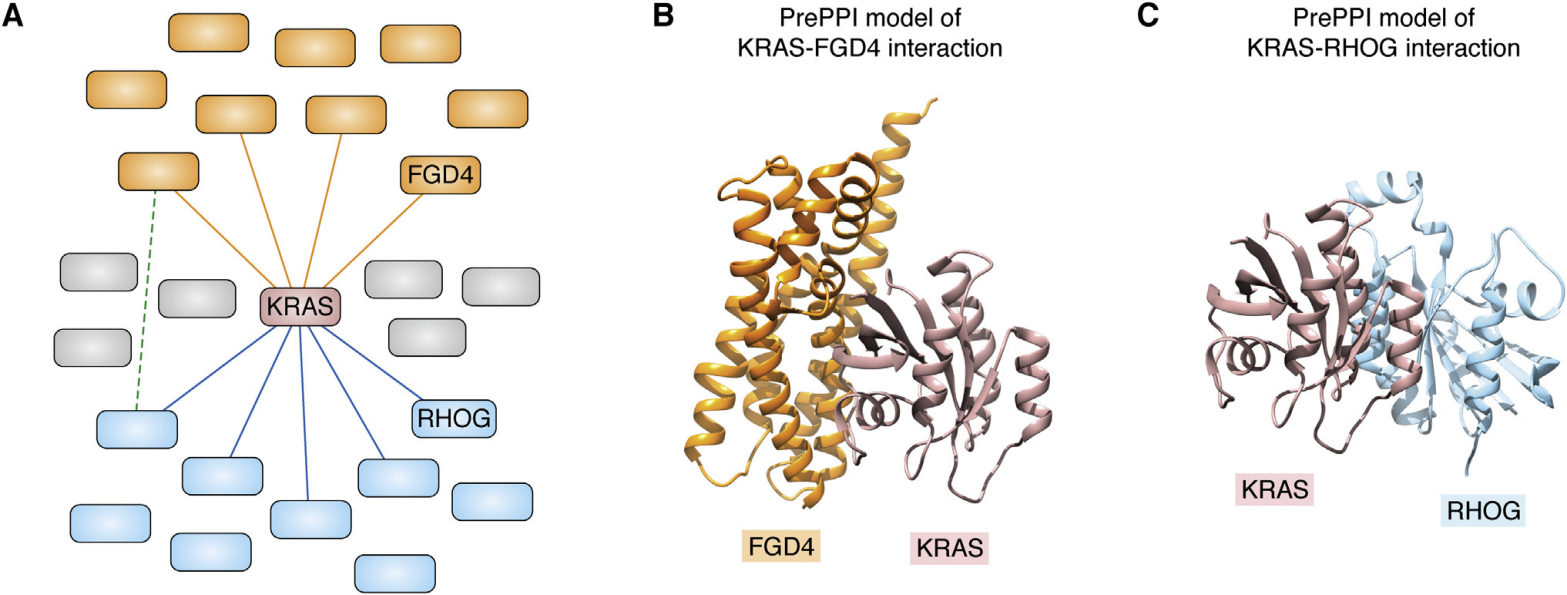
**Aspects of the lung adenocarcinoma (LUAD)–specific KRas SigMap.***A,* schematic diagram of the OncoSig SigMap. For ease of depiction, only a few nodes are drawn for the highest scoring SigMap members; in practice, there are 250 top-scoring (false positive rate <0.01) members of the LUAD-specific KRas SigMap. KRas is denoted as the central node (KRAS, *rose*). *Orange nodes* represent upstream regulators of KRas, and *blue nodes* represent downstream effectors of KRas. *Orange* and *blue lines* denote PrePPI-predicted physical interactions between KRas and upstream and downstream proteins. *Gray nodes* represent PrePPI physical interactors of KRas that do not have associated upstream or downstream predictions. The *dotted green line* denotes predicted regulatory interactions between proteins upstream and downstream of KRas. PrePPI-predicted KRas interactions with the FYVE, RhoGEF, and PH domain–containing protein 4 (FDG4, *orange*) and RhoG (RHOG, *blue*) are highlighted. *B,* PrePPI model of the interaction between KRas (*rose*) and the GEF domain of FGD4 (*orange*), a predicted upstream activator in the LUAD SigMap. *C,* PrePPI model of the interaction between KRas (*rose*) and RhoG (*blue*), a predicted downstream effector in the LUAD SigMap. RHOG was validated as synthetic lethal with KRAS^mut^ in LUAD spheroids (27). In (*B*) and (*C*), KRas is in a similar orientation to facilitate the comparison of the binding modes of FGD4 and RhoG. The presence of FGD4 and RhoG in the KRas SigMap implicates KRas in cytoskeletal processes and cell migration in the LUAD context.

Networks derived from pairwise-interaction assays or computational predictions generally neither account for nor discriminate between cellular contexts (61). Recent approaches have started to address the challenge of “context-specific interactions” by incorporating cell line-, tumor-, or tissue-specific information (62, 63, 64, 65, 66). However, comprehensive proteome-wide depiction of human interactomes across different tissue contexts remains elusive. To address these challenges, we developed an integrative ML framework (OncoSig) using PrePPI and other computationally derived interactomes for the systematic, *de novo* reconstruction of tumor-specific molecular-interaction signaling maps (SigMaps), anchored on any oncoprotein of interest (27). Specifically, as illustrated in Figure 3, an oncoprotein-specific SigMap recapitulates the molecular architecture necessary to functionally modulate and mediate its activity within a specific cellular context, including its physical cognate binding partners.

OncoSig infers context-specific SigMaps by integrating PrePPI with complementary evidence from transcriptional and post-translational interactions from gene expression and mutational profiles from TCGA. PrePPI provides context-independent and structure-based information on the “reference” human protein interactome. ARACNe (67, 68), VIPER (69), and CINDy (70, 71) provide information from genomic data, including, as depicted in Figure 3*A*, upstream modulators (*orange*) and downstream effectors (*blue*) of a protein of interest (*rose*) and regulatory interactions, such as feedback loops, among them (*green dotted line*). They further account for tumor specificity since they are based on the analysis of molecular profile data from patient samples corresponding to different TCGA tumor types (*e.g.*, lung adenocarcinoma or colon adenocarcinoma). The SigMap generated for lung adenocarcinoma recapitulated published KRas biology and identified novel KRas-associated proteins whose genes were experimentally validated as synthetic lethal with KRAS^mut^ in 3D spheroid models derived from primary lung cancer cells (27).

**Figure 4:**
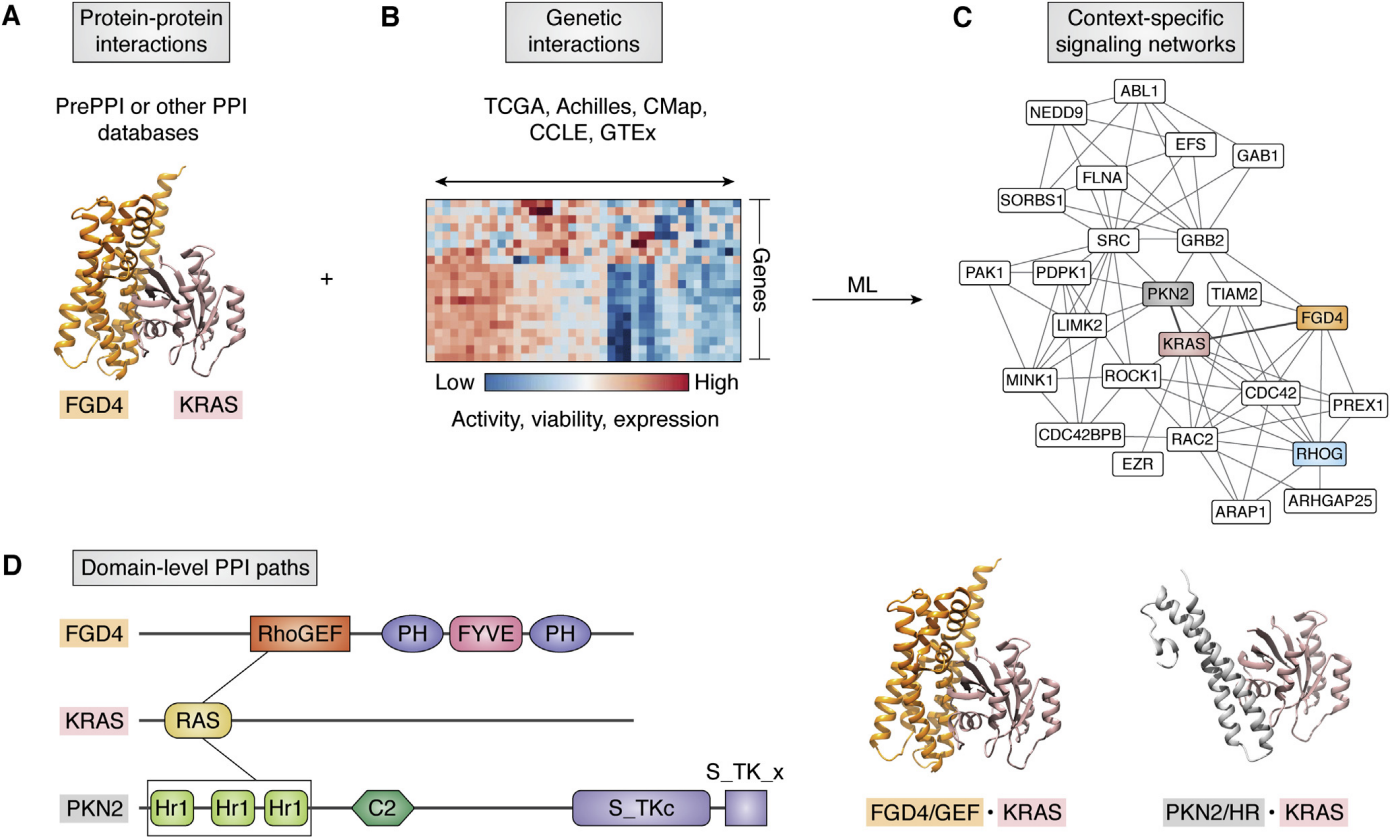
**Creating context-dependent SigNets.***A,* physical interactome: input from a physical interactome is represented by the PrePPI predicted model for the FGD4/KRas complex. *B,* nonstructural interactome: evidence from genetic screens is orthogonal (complementary) to physical PPIs and provides information on context dependency. *C,* machine learning using data from (*A*) and (*B*) with training on a gold standard set representing, for example, proteins known to be involved in a signaling pathway or a cellular process, ranks or prioritizes proteins specific to the given context defined in (*B*). Combining (*A*) and (*B*) can then be used to produce a context-dependent network of physical PPIs. Shown are PrePPI interactions among a subset of top-scoring proteins in the lung adenocarcinoma–specific KRas SigMap (from Fig. 3) that are enriched for the biological process “actin cytoskeleton reorganization.” *D,* one physical path from (*C*) is comprised of consecutive PPIs—FGD4 (*orange*)/KRas (*rose*) and KRas/Ser-Thr Protein kinase N2 (PKN2, *gray*)—that are described at the linear domain level (*left*) and structural models of interacting domains (*right*).

Increasingly, PPIs in existing networks are inferred from genetic interactions, which are typically based on the correlation of gene profiles across many conditions (*e.g.*, cell lines or drug treatments) (19). While protein complexes are enriched in genetic interactions (72, 73), genetic interactions do not necessarily correspond to physical PPIs and, thus, serve as an orthogonal and complementary resource for direct physical PPIs as contained, for example, in the PrePPI database. Thus, in parallel to the development of OncoSig where PrePPI was integrated with genetic interactions derived from TCGA, context-specific PPI networks (or SigNets) can be obtained by integrating physical protein interactomes with genetic interactions based on gene profiles derived from HT genomic screens of human cancer cell lines (23, 74). Figure 4 illustrates a generalized scheme to derive context-dependent SigNets. Of note, Figure 4*D* highlights the description of individual pathways at the level of interactions between individual protein domains.

## Structural systems pharmacology

Systems pharmacology approaches typically aim to leverage network topology to elucidate drug mechanism of action, discover new targets, and design combination therapies (75). This has been made possible through the integration of omics technologies with large-scale chemical compound repositories and databases of drug–protein interactions and bioactivity data (76, 77, 78, 79, 80, 81), Moreover, the application of HT screening and sequencing technologies at the single patient level has facilitated the application of systems pharmacology in precision medicine (“N-of-1”) contexts (82, 83, 84). Systems pharmacology thus leverages network-based perspectives of human disease in next-generation drug discovery.

While the intersection of network analysis and phenotypic screens has proved powerful, systems-level implementation of traditional drug discovery tools is necessary for maximum impact. For example, if a new target is identified *via* network analysis, it is then necessary to find a compound that effectively and specifically inhibits that target. Or, if a particular drug is found to be effective in a phenotypic screen, in many cases, it will be necessary to identify the actual target(s). Furthermore, although drug repurposing has yielded important discoveries, the continuing exploration of chemical space is clearly of great importance.

Traditional drug discovery has relied on both cheminformatic tools and protein structure–based tools. The former is ultimately based on the assumption that chemically similar ligands will bind to similar proteins (*e.g.*, (85, 86)). Numerous tools are available to represent chemicals as molecular fingerprints in a format that can be used for rapid similarity searches based, for example, on Tanimoto coefficients (87). The Similarity Ensemble Approach (SEA) uses this principle to relate proteins based on the ligands they bind and, thus, identifies new protein targets for existing drugs (88). ML is playing an increasingly important role in this area where, in effect, pairwise chemical similarity relationships are supplanted by “learning” what compounds might target a particular protein or have a desired biological effect as determined by training data obtained from aptly designed HT screens (89).

The most common current uses of protein structure are in ligand docking and lead optimization, and significant advances continue to be made in both these technologies. For example, flexible docking helps escape the constraint of using rigid protein structures (90), and neural networks have been trained to score docking poses (91). In the area of lead optimization, free energy perturbation methods can yield truly accurate relative binding free energies of a congeneric series of compounds (92, 93), although accuracy inevitably is compromised if a homology model rather than a crystal structure of the protein–ligand complex is used. Algorithmic advances combined with high performance computing, and particularly the use of Graphical Processing Units (GPUs), have enabled the ever-expanding use of these tools, but there are still limitations for their use on a true genome-wide scale.

Our group and others are developing alternate approaches that leverage the Structural Blast concept. Similar to what has been described previously for PPIs, these methods exploit available structural information under the assumption that structural similarities between proteins provide clues as to what compounds will bind a protein and where. One approach is to align entire protein structures or substructures to PDB protein–compound complexes, which have the effect of moving the ligand in a template structure into the coordinate system of the query protein structure (94) (Fig. 1*C*). The resulting ligand–protein interaction model can then be scored by enumerating the physiochemical features of the predicted binding site. An alternative approach is to search for regions in potential target proteins that structurally align to binding pockets in PDB complexes (*e.g.*, (95, 96)).

Structural alignment is a way to explore protein structure space, whereas chemical similarity searches enable the exploration of chemical space. A number of efforts to combine the two have been described (95, 96) where the link is a PDB complex. For example, one can start with a query compound identified in a phenotypic screen, search for chemically similar compounds in a database of PDB complexes, and then use structural alignment to identify other proteins that might bind to the original compound. In parallel, starting with a target protein, structural alignment can be used to identify related proteins in PDB complexes, and then chemical similarity can be used to identify lead compounds that bind to the original query protein. This low-resolution strategy, when combined with a battery of docking and lead optimization technologies, offers the possibility of true genome-wide structure-based prediction of ligand–protein interactions.

## Concluding remarks

We have highlighted a daunting array of genomic technologies and databases that have emerged in the past few years and that offer the possibility of transforming both basic and translational biomedical research. Given the proper tools, we have argued that the strategy of exploiting the information available in the PDB can make this database *the* critical resource that enables the integration of structural biology with systems biology. We are now in a position to create and probe tissue- and disease-specific structure–informed protein interaction networks and similar networks that describe pathogen infection. The integration of structure in these networks is the only way to gain mechanistic insights and to link these networks to drug discovery tools, which themselves are undergoing rapid evolution. As the information available in the PDB grows, the ways in which that information can be used to carry out systems-wide analysis of biological processes will grow as well.

## Conflict of interest

The authors declare that they have no conflicts of interest with the contents of this article.
